# Pathophysiological and diagnostic importance of fatty acid-binding protein 1 in heart failure with preserved ejection fraction

**DOI:** 10.1038/s41598-021-00760-2

**Published:** 2021-10-27

**Authors:** Tomonari Harada, Takeshi Araki, Hiroaki Sunaga, Kazuki Kagami, Kuniko Yoshida, Toshimitsu Kato, Ryo Kawakami, Junichi Tomono, Naoki Wada, Tatsuya Iso, Masahiko Kurabayashi, Masaru Obokata

**Affiliations:** 1 Department of Cardiovascular Medicine, Gunma University Graduate School of Medicine, 3-39-22 Showa-machi, Maebashi, Gunma 371-8511 Japan; 2 Center for Liberal Arts and Sciences, Ashikaga University, Ashikaga, Tochigi Japan; 3 Division of Cardiovascular Medicine, National Defense Medical College, Tokorozawa, Saitama Japan; 4 Department of Rehabilitation Medicine, Gunma University Graduate School of Medicine, Maebashi, Gunma Japan

**Keywords:** Biomarkers, Cardiology

## Abstract

Elevated intracardiac pressure at rest and/or exercise is a fundamental abnormality in heart failure with preserved ejection fraction (HFpEF). Fatty acid-binding protein 1 (FABP1) is proposed to be a sensitive biomarker for liver injury. We sought to determine whether FABP1 at rest would be elevated in HFpEF and would correlate with echocardiographic markers of intracardiac pressures at rest and during exercise. In this prospective study, subjects with HFpEF (n = 22) and control subjects without HF (n = 23) underwent resting FABP1 measurements and supine bicycle exercise echocardiography. Although levels of conventional hepatic enzymes were similar between groups, FABP1 levels were elevated in HFpEF compared to controls (45 [25–68] vs. 18 [14–24] ng/mL, *p* = 0.0008). FABP1 levels were correlated with radiographic and blood-based markers of congestion, hemodynamic derangements during peak exercise (E/e’, r = 0.50; right atrial pressure, r = 0.35; pulmonary artery systolic pressure, r = 0.46), reduced exercise cardiac output (r = − 0.49), and poor exercise workload achieved (r = − 0.40, all *p* < 0.05). FABP1 distinguished HFpEF from controls with an area under the curve of 0.79 (*p* = 0.003) and had an incremental diagnostic value over the H_2_FPEF score (*p* = 0.007). In conclusion, FABP1 could be a novel hepatic biomarker that associates with hemodynamic derangements, reduced cardiac output, and poor exercise capacity in HFpEF.

## Introduction

Approximately half of the patients with heart failure (HF) have a preserved ejection fraction (HFpEF)^1^. Increases in cardiac filling pressures are a fundamental hemodynamic abnormality in patients with HFpEF and high filling pressures are associated with symptoms of dyspnea, poor aerobic capacity, and worse clinical outcomes^2,3^. In patients with HFpEF, left ventricular (LV) filling pressures are often normal at rest, but become abnormally increased only during physiological stress such as exercise^4,5^. Recent studies have demonstrated the importance of exercise stress testing such as diastolic stress echocardiography to unmask hemodynamic abnormalities during exercise and to enhance the diagnosis of HFpEF^6–10^.

Natriuretic peptides are the most commonly used biomarker to help the diagnosis of HFpEF and are endorsed in consensus guidelines to facilitate the diagnosis of HFpEF^7,11,12^. A number of other candidate biomarkers that may relate to the pathophysiology of HFpEF have been reported, including myocardial injury and stress, neurohormonal activation, inflammation, and metabolism^13–16^. However, very little data are available regarding the biomarkers representing the cardio-hepatic interactions. Fatty acid-binding protein 1 (FABP1), also known as liver-FABP, is 14.4-kDa protein, which is primarily expressed in the liver^17,18^. Circulating FABP1 levels are induced by liver injuries such as acute and chronic hepatitis, liver cirrhosis, and nonalcoholic steatohepatitis^19–21^. Emerging data have suggested that FABP1 is a sensitive marker to detect early liver injury^22^. Our group demonstrated that FABP1 levels were elevated in HF patients compared to controls and predicted poor clinical outcomes^23^.

Given this background, we hypothesized that FABP1 levels at rest would be elevated in patients with HFpEF compared to control subjects free of HF and that the magnitude of elevation would correlate with hemodynamic perturbations at rest and during exercise in HFpEF. We also hypothesized that FABP1 would have an incremental diagnostic value to identify HFpEF over the validated H_2_FPEF score.

## Results

### Baseline characteristics

We enrolled 23 control subjects and 22 HFpEF patients in this study. Compared to control subjects, patients with HFpEF were older and had radiographic and blood-based signs of congestion, evidenced by a higher prevalence of cardiomegaly and greater N-terminal pro-B-type natriuretic peptide (NT-proBNP) levels (Table 1). Sex, body mass index, and prevalence of comorbidities did not differ in HFpEF and control subjects. Pulmonary rales and peripheral edema were rare in both groups. The use of cardiovascular medications was similar between groups. As expected, the H_2_FPEF score was higher in patients with HFpEF than controls. Hemoglobin and creatinine levels were similar between HFpEF patients and controls.

**Table 1:** Baseline Characteristics.

	Controls (n = 23)	HFpEF (n = 22)	*p* value
Age (years)	63 ± 12	75 ± 7	0.0002
Female, n (%)	14 (61%)	13 (59%)	0.90
Body mass index (kg/m^2^)	24.0 ± 4.3	22.9 ± 3.9	0.39
**Comorbidities**			
Coronary disease, n (%)	1 (4%)	2 (9%)	0.52
Diabetes mellitus, n (%)	1 (4%)	5 (23%)	0.07
Hypertension, n (%)	17 (74%)	18 (82%)	0.52
Atrial fibrillation, n (%)	2 (9%)	7 (32%)	0.05
Interstitial pneumonia, n (%)	4 (17%)	4 (18%)	0.94
**Physical examination**			
Rales, n (%)	1 (4%)	0 (0%)	0.32
Edema (none/1 + /2 +), (%)	70%/22%/8%	77%/23%/0%	0.37
**Chest radiography**			
Cardiothoracic ratio, %	52 ± 6	55 ± 8	0.12
Cardiomegaly, n (%)	11 (48%)	18 (86%)	0.008
Pleural effusion, n (%)	0 (0%)	1 (4%)	0.30
**Medications**			
ACEI or ARB, n (%)	10 (43%)	7 (32%)	0.42
Beta-blocker, n (%)	4 (17%)	6 (27%)	0.43
Loop diuretics, n (%)	6 (26%)	6 (27%)	0.93
H_2_FPEF score	2 (1, 3)	3 (2, 4)	0.01
**Laboratories**			
Hemoglobin (g/dL)	13.2 ± 1.2	12.6 ± 1.7	0.23
NT-proBNP (pg/mL)	84 (53, 111)	257 (129, 820)	< 0.0001
AST (U/L)	22 (17, 24)	25 (19, 27)	0.14
ALT (U/L)	17 (12, 21)	14 (12, 17)	0.18
γGT (U/L)	22 (16, 29)	18 (12, 32)	0.45
ALP (U/mL)	217 (171, 271)	199 (186, 238)	0.81
T-bilirubin (mg/dL)	0.6 (0.5, 0.7)	0.8 (0.6, 1.0)	0.08
Creatinine (mg/dL)	0.9 ± 0.4	0.9 ± 0.5	0.56
FABP1 (ng/mL)	18 (14, 24)	45 (25, 68)	0.0008

Data are mean ± SD, median (interquartile range), or n (%). ACEI, angiotensin-converting enzyme inhibitors; ALP, alkaline phosphatase; ALT, alanine transaminase; ARB, angiotensin-receptor blockers; AST, aspartate transaminase; NT-proBNP, N-terminal pro-B-type natriuretic peptide; FABP, fatty acid-binding protein; HFpEF, heart failure with preserved ejection fraction; T-bilirubin, total bilirubin; and γGT, γ-glutamyl transferase.

**Figure 1:**
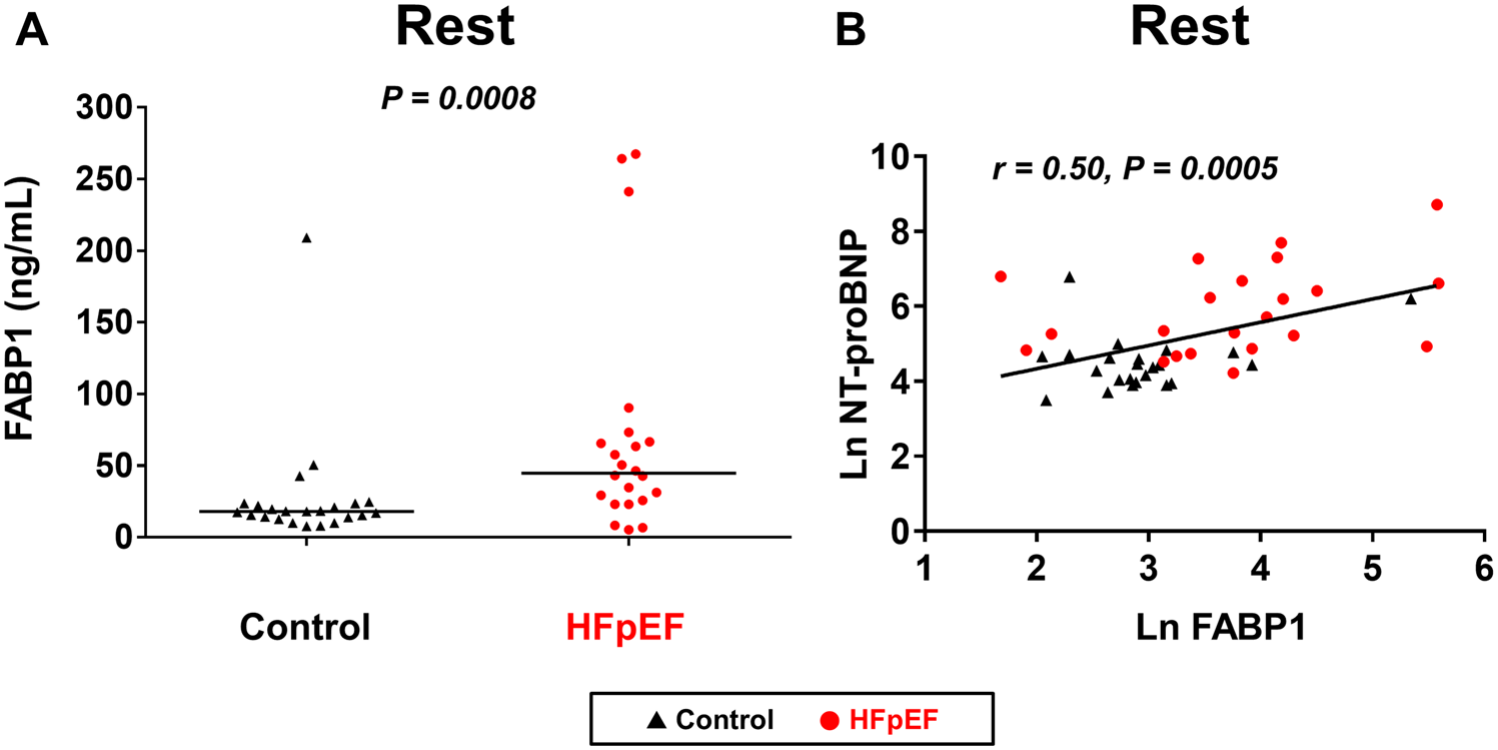
(**A**) Compared with control subjects, serum fatty acid-binding protein 1 (FABP1) levels at rest were elevated in patients with heart failure with preserved ejection fraction (HFpEF). (**B**) Levels of FABP1 at rest were moderately correlated with serum levels of N-terminal pro-B-type natriuretic peptide (NT-proBNP).

Conventional hepatobiliary markers were on average within the normal range in patients with HFpEF and were similar between groups. However, FABP1 levels were elevated in patients with HFpEF compared to controls (Fig. 1A). The difference remained significant after adjusting for either age (*p* = 0.04) or any hepatobiliary enzymes (all *p* < 0.05). FABP1 levels were not correlated with conventional hepatobiliary enzymes (all *p* > 0.15). In contrast, FABP1 levels were directly correlated with radiographic and blood biomarkers of congestion (cardiothoracic ratio, r = 0.33, *p* = 0.03; and NT-proBNP levels, r = 0.50, *p* = 0.0005, Fig. 1B).

### Baseline LV function, hemodynamics, and their relationships with FABP1

**Figure 2:**
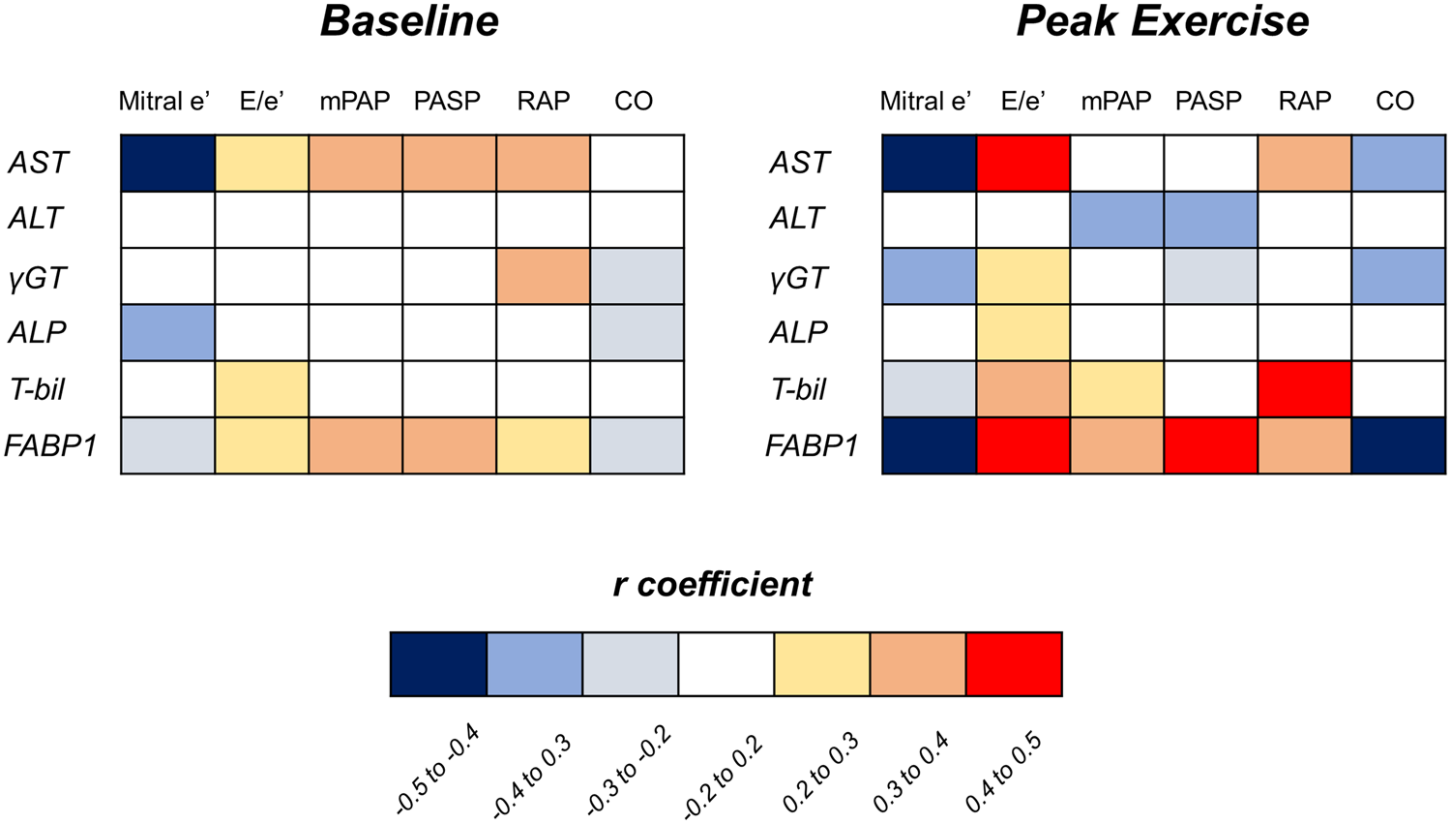
Heatmap showing relationships between resting biomarker levels and echocardiographic marker of left ventricular diastolic function, pulmonary artery pressures, and cardiac output at rest and during peak exercise. Biomarker levels except for total bilirubin were log-transformed. ALP, alkaline phosphatase; ALT, alanine transaminase; AST, aspartate transaminase; CO, cardiac output; E/e’, the ratio of early diastolic mitral inflow velocity to mitral annular tissue velocity; mPAP, pulmonary artery mean pressure; PASP, pulmonary artery systolic pressure; RAP, right atrial pressure; T-bil, total bilirubin; γGT, γ-glutamyl transferase; and other abbreviations as in Fig. 1.

At rest, heart rate, systolic blood pressure (BP), and oxygen saturation were similar between HFpEF and control subjects (Table 2). Compared to controls, patients with HFpEF displayed greater LV size and mass, larger left atrial (LA) volume, lower EF and mitral e’ velocity, higher mitral E velocity and the ratio of E to mitral annular e’ velocity (E/e’) ratio, and reduced cardiac output (CO). As compared to control subjects, estimated pulmonary artery (PA) and right atrial (RA) pressures were elevated in patients with HFpEF, whereas right ventricular (RV) systolic function (tricuspid annular plane systolic excursion [TAPSE] and systolic tissue velocities at the lateral tricuspid annulus [TV s’]) was not different at rest. Levels of FABP1 at rest were correlated with resting mean PA pressure (mPAP)(r = 0.38, *p* = 0.01) and PA systolic pressure (PASP)(r = 0.38, *p* = 0.01) while there were marginal correlations with E/e’ ratio and RA pressure (both r = 0.29, *p* = 0.05) (Fig. 2). Serum aspartate transaminase (AST) was also correlated with mitral e’ velocity (r = − 0.46, *p* = 0.0003), mPAP (r = 0.34, *p* = 0.03), PASP (r = 0.34, *p* = 0.03), and RAP (r = 0.36, *p* = 0.02) although its circulating levels were similar in HFpEF and controls (*p* = 0.14).

**Table 2:** Baseline Echocardiographic Data.

	Controls (n = 23)	HFpEF (n = 22)	*p* value
Heart rate (bpm)	80 ± 16	72 ± 14	0.06
Systolic BP (mmHg)	128 ± 21	130 ± 21	0.78
Saturation (%)	96 ± 2	97 ± 1	0.17
LV diastolic dimension (mm)	42 ± 4	46 ± 6	0.02
LV mass index (g/m^2^)	78 ± 17	91 ± 17	0.02
LV ejection fraction (%)	66 ± 8	61 ± 8	0.04
LA volume index (mL/m^2^)	24 ± 8	45 ± 22	0.0002
E-wave (cm/s)	65 ± 21	89 ± 35	0.01
A-wave (cm/s)	78 ± 25	94 ± 26	0.05
Mitral e’ (cm/s)	6.7 ± 1.6	5.6 ± 1.6	0.03
Mitral s’ (cm/s)	8.6 ± 2.0	6.4 ± 1.7	0.0002
E/e’ ratio	10 ± 3	17 ± 10	0.002
Cardiac output (L/min)	4.9 ± 1.6	4.0 ± 0.9	0.03
TAPSE (mm)	17 ± 5	17 ± 5	0.76
TV s’ (cm/s)	13 ± 4	12 ± 3	0.35
mPAP (mmHg)	13 ± 4	18 ± 5	0.001
PASP (mmHg)	18 ± 6	25 ± 9	0.002
RAP (mmHg)	3 ± 1	5 ± 4	0.03

Data are mean ± SD, or median (interquartile range). BP, blood pressure; E/e’ ratio, the ratio of early diastolic mitral inflow to mitral annular tissue velocities; LA, left atrial; LV, left ventricular; mPAP, mean pulmonary artery pressure; PASP, pulmonary artery systolic pressure; RAP, right atrial pressure; TAPSE, tricuspid annular plane systolic excursion; TV, tricuspid valve; and other abbreviations as in Table 1.

### LV function, hemodynamics, and their relationships with FABP1 during exercise

**Figure 3:**
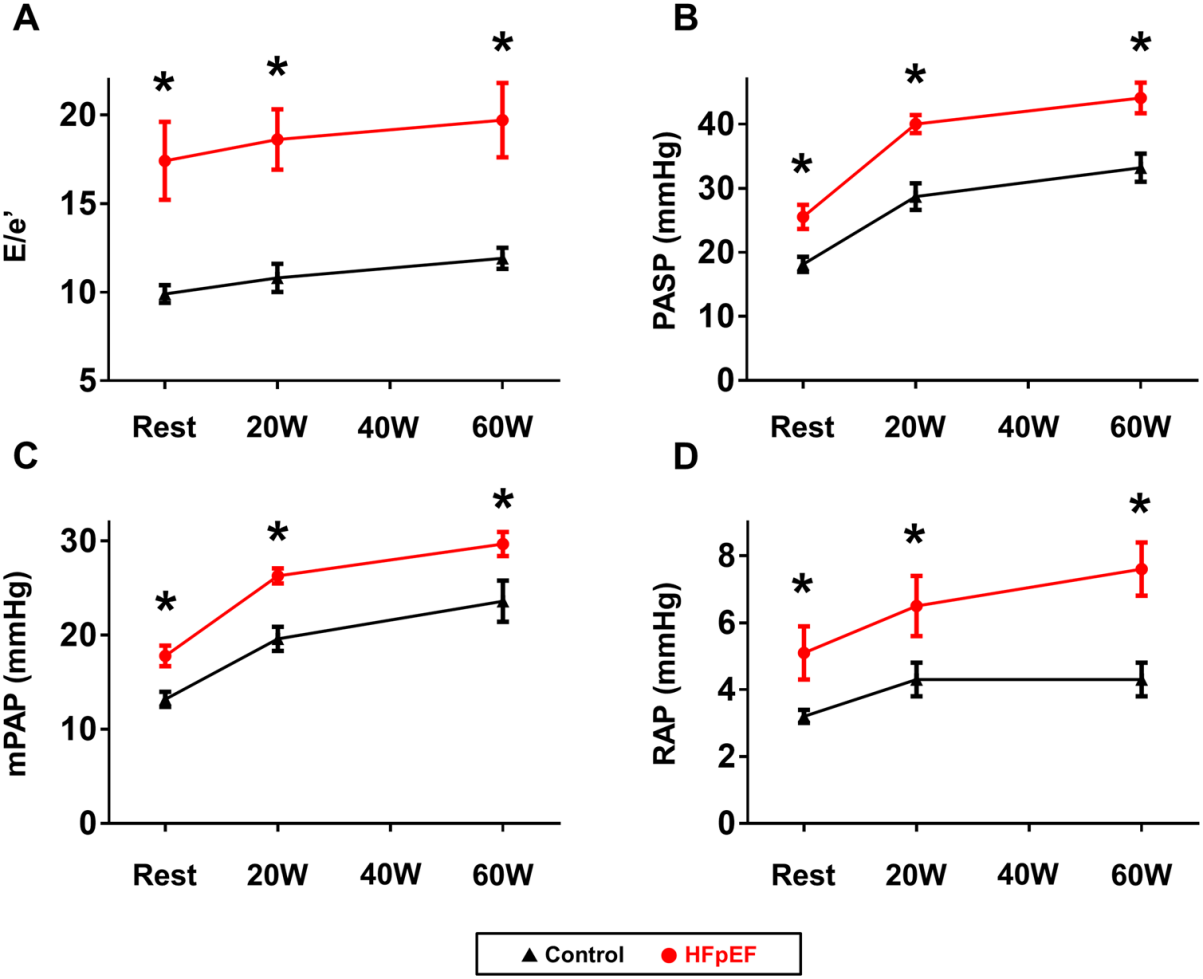
E/e’ ratio, PASP, mPAP, and RAP as a function of workload in patients with HFpEF and control subjects. Abbreviations as in Figs. 1 and 2. *p < 0.05 versus control subjects at each stage.

**Figure 4:**
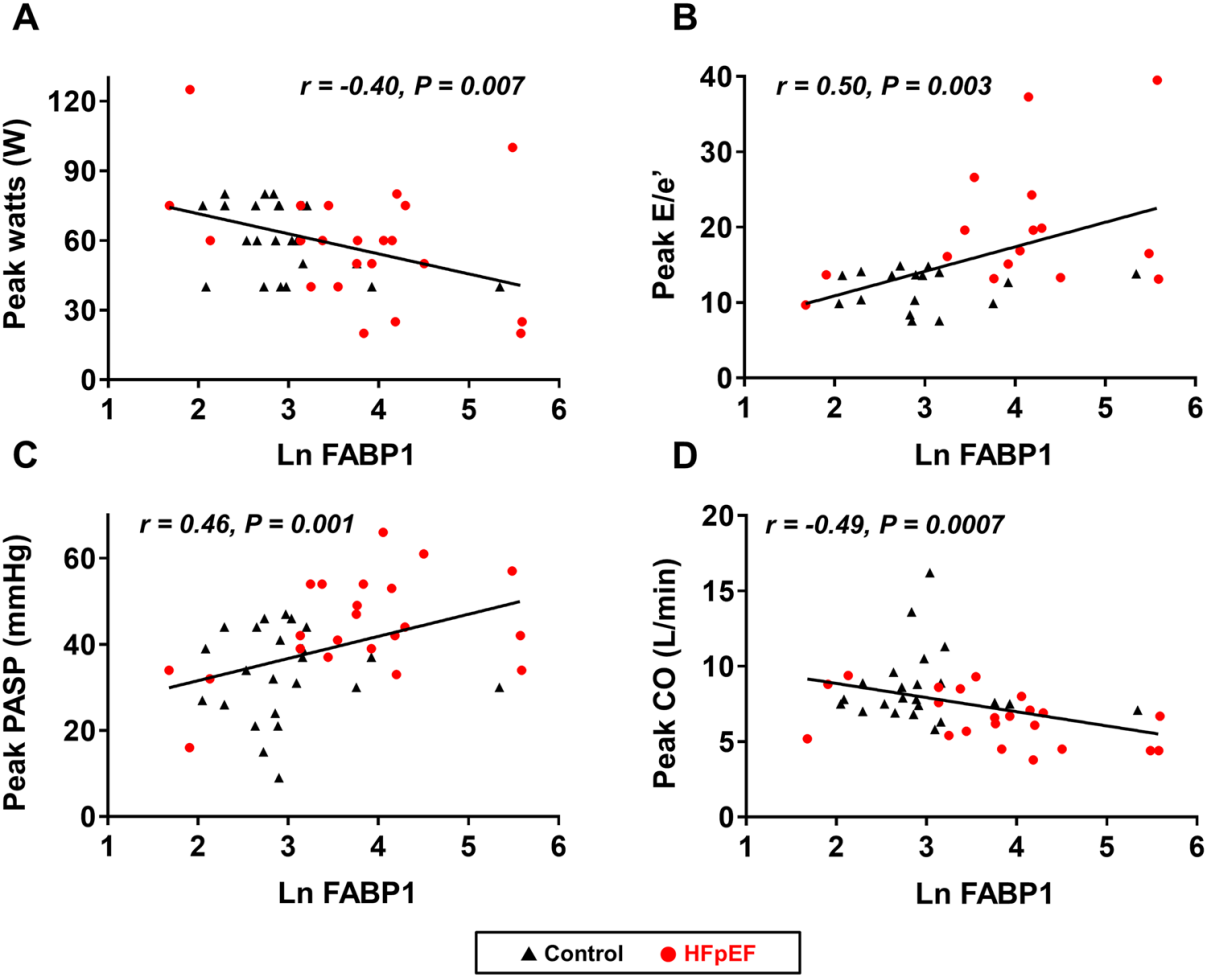
Levels of FABP1 were correlated with exercise intolerance reflected by lower peak watts achieved, higher E/e’ ratio and PASP, and lower CO during peak exercise. Abbreviations as in Figs. 1 and 2.

With low level (20 W) and peak exercise, heart rate, systolic BP, and oxygen saturation were similar between groups (Table 3). Compared to control subjects, mitral E velocity, E/e’ ratio, PA pressures, and RA pressure were higher and mitral e’ and s’ velocities were again lower in HFpEF subjects during 20 W and peak exercise (Fig. 3). Subjects with HFpEF displayed lower CO during peak exercise than controls. Levels of FABP1 at rest were correlated with poor exercise capacity as reflected by lower peak watts achieved and shorter exercise duration (r = − 0.40, *p* = 0.007 and r = − 0.34, *p* = 0.03). Resting FABP1 levels were also correlated with poorer LV diastolic reserve (e’, r = − 0.46, *p* = 0.007), higher LV filling pressure (E/e’ ratio; r = 0.50, *p* = 0.003), more severe pulmonary hypertension and RA hypertension (mPAP; r = 0.34, *p* = 0.03; PASP, r = 0.46, *p* = 0.001; and RAP, r = 0.35, *p* = 0.02), and lower CO (r = − 0.49, *p* = 0.0007) during peak exercise (Figs. 2, 4). Sensitivity analysis performed in HFpEF patients only showed similar relationships of FABP1 with exercise capacity and hemodynamics although some associations were not statistically significant possibly due to smaller sample size (exercise workload achieved, r = − 0.41, *p* = 0.06; NT-proBNP levels, r = 0.39, *p* = 0.08; exercise PASP, r = 0.43, *p* = 0.04; exercise CO, r = − 0.48, *p* = 0.02; and exercise E/e’, r = 0.36, *p* = 0.11). Modest correlations were observed between other hepatobiliary markers and echocardiographic parameters during peak exercise among all participants (Fig. 2).

**Table 3:** Echocardiographic Data during Exercise.

	Controls (n = 23)	HFpEF (n = 22)	*p* value
**20 W Exercise**			
Heart rate (bpm)	95 ± 15	93 ± 18	0.64
Systolic BP (mmHg)	148 ± 26	143 ± 26	0.50
Saturation (%)	94 ± 3	95 ± 3	0.56
LV ejection fraction (%)	69 ± 7	65 ± 9	0.05
E-wave (cm/s)	91 ± 24	117 ± 30	0.003
A-wave (cm/s)	94 ± 21	107 ± 28	0.12
Mitral e’ (cm/s)	8.7 ± 2.4	6.5 ± 1.6	0.0008
Mitral s’ (cm/s)	8.3 ± 1.8	6.7 ± 1.5	0.003
E/e’ ratio	11 ± 4	19 ± 8	< 0.0001
Cardiac output (L/min)	6.6 ± 1.8	5.7 ± 1.4	0.07
TAPSE (mm)	20 ± 4	18 ± 5	0.26
TV s’ (cm/s)	14 ± 3	13 ± 3	0.45
mPAP (mmHg)	20 ± 6	26 ± 4	0.0001
PASP (mmHg)	29 ± 10	40 ± 6	< 0.0001
RAP (mmHg)	4 ± 2	6 ± 4	0.03
**Peak Exercise**			
Peak watts (W)	60 (40, 75)	60 (40, 75)	0.50
Exercise time (min)	10.6 ± 2.7	9.9 ± 2.5	0.36
Heart rate (bpm)	117 ± 20	112 ± 25	0.44
Systolic BP (mmHg)	169 ± 29	160 ± 24	0.26
Saturation (%)	93 ± 5	94 ± 3	0.48
LV ejection fraction (%)	73 ± 8	69 ± 12	0.21
E-wave (cm/s)	113 ± 21	133 ± 30	0.02
A-wave (cm/s)	115 ± 30	118 ± 40	0.81
Mitral e’ (cm/s)	9.7 ± 2.5	7.5 ± 1.9	0.002
Mitral s’ (cm/s)	9.6 ± 2.4	7.4 ± 1.9	0.001
E/e’ ratio	12 ± 3	20 ± 9	0.001
Cardiac output (L/min)	8.6 ± 2.4	6.6 ± 1.7	0.002
TAPSE (mm)	20 ± 5	18 ± 5	0.21
TV s’ (cm/s)	15 ± 3	14 ± 4	0.44
mPAP (mmHg)	24 ± 6	30 ± 6	0.001
PASP (mmHg)	33 ± 11	44 ± 11	0.002
RAP (mmHg)	4 ± 2	8 ± 4	0.002

Data are mean ± SD, or median (interquartile range). Abbreviations as in Table 1 and 2.

### Diagnostic performance of FABP1

As expected, The H_2_FPEF score and NT-proBNP levels demonstrated good discriminatory abilities for identifying HFpEF, with areas under the curve (AUCs) of 0.72 and 0.88 (*p* = 0.009 and *p* < 0.0001), respectively. FABP1 distinguished HFpEF from control subjects with an AUC of 0.79 (*p* = 0.003) whereas other hepatobiliary markers did not (Table 4). FABP1 had an incremental diagnostic value over the H_2_FPEF score (global chi-square 14.1 vs. 6.9, *p* = 0.007).

**Table 4:** Diagnostic Performance of Biomarkers to Diagnose HFpEF.

	AUC	*p* value
NT-proBNP (pg/mL)	0.88	< 0.0001
AST (U/L)	0.63	0.24
ALT (U/L)	0.62	0.28
γGT (U/L)	0.57	0.68
ALP (U/mL)	0.52	0.90
T-bilirubin (mg/dL)	0.66	0.05
FABP1 (ng/mL)	0.79	0.003

AUC, area under the curve; and other abbreviations as in Table 1.

### Sensitivity analyses

Of the 45 participants, liver sonographic examinations that were performed within a year from exercise echocardiography were available in six patients. Of the six patients, two patients were found to have mild chronic hepatitis and one had mild fatty liver while no evidence of acute or chronic hepatitis was observed in the others. Sensitivity analyses were then performed excluding the three patients with liver diseases. We found that (1) FABP1 levels remained significantly higher in HFpEF patients than controls (46 [24, 70] ng/mL in HFpEF [n = 21] vs. 17 [13, 23] ng/mL in controls [n = 21], *p* = 0.0009); (2) FABP1 remained significantly associated with NT-proBNP levels (r = 0.50, *p* = 0.0007), exercise E/e’ (r = 0.48, *p* = 0.002), exercise eRAP (r = 0.38, *p* = 0.02), exercise PASP (r = 0.40, *p* = 0.008), exercise CO (r = − 0.40, *p* = 0.009), and exercise workload achieved (r = − 0.38, *p* = 0.01); and (3) FABP1 could distinguish HFpEF from controls with an AUC of 0.80 (*p* = 0.002), with an incremental diagnostic value over the H_2_FPEF score (global chi square 12.3 vs. 4.5, *p* = 0.006).

## Discussion

We demonstrated, for the first time to our knowledge, the robust relationships between serum FABP1 and echocardiographic measures characterizing HFpEF. We found that FABP1 but not conventional hepatobiliary markers was significantly elevated at rest in patients with HFpEF compared to controls. Interestingly, FABP1 levels were associated with markers of congestion and alteration of parameters for systolic and diastolic reserve, biventricular filling pressures, pulmonary hypertension, and CO during exercise in HFpEF. Additionally, FABP1 levels were associated with the presence of HFpEF, with an incremental diagnostic value over the H_2_FPEF score. Given that circulating FABP1 is most exclusively derived from the liver, our data suggest that FABP1 could be a novel hepatic biomarker that associates with hemodynamic derangements, lower cardiac output, and reduced exercise capacity in HFpEF.

### Potential mechanisms for FABP1 elevation in HFpEF

Biomarkers provide valuable information to understand the specific pathophysiological pathways that relate to the disease^24^. FABPs are relatively small cytoplasmic proteins (14–15 kDa) abundantly expressed in a tissue-restricted manner; therefore, in response to tissue injury, FABPs diffuse more rapidly through the interstitial space and the endothelial clefts to circulation than large proteins such as alanine transaminase (ALT) (96 kDa) or AST (90 kDa)^25^. As such, FABP1 could serve as a biomarker for an earlier phase of hepatic injury where conventional liver markers are not released. In keeping with this notion, our data showed that circulating FABP1 levels were increased in the absence of elevation of AST and ALT. Accordingly, one of the most likely mechanisms for FABP1 elevation in HFpEF patients is a response to early or minimal hepatic injury.

However, our simple correlation analyses revealed no significant correlation between FABP1 and AST, ALT, or the other conventional hepatic enzymes, arguing against the hepatic injury as a sole mechanism of an increase in circulating FABP1 in HFpEF. We recently found that FABP1 levels were significantly increased during exercise and were significantly correlated with plasma norepinephrine levels in healthy volunteers (manuscript in preparation). These results suggest that exercise induces circulating FABP1 through the mechanisms involving sympathetic nervous system activation. Thus, it is intriguing to speculate that an elevation of FABP1 in HFpEF is partly due to a hepatic activation of adrenergic signaling. Further studies are needed to determine the mechanisms underlying elevation in FABP1 in patients with HFpEF.

### A potential link between FABP1, hepatic injury, and hemodynamic derangements during exercise in HFpEF

HFpEF is a clinical syndrome that can be characterized by reduced cardiovascular reserve which leads to an elevation in LV filling pressure and secondary pulmonary hypertension during exercise^2,26^. The current study demonstrated correlations of FABP1 levels with radiographic and blood markers of congestion and echocardiographic evidence of hemodynamic derangements (higher E/e’ ratio, PASP, and eRAP during peak exercise). The cross-sectional design of our study cannot determine whether hemodynamic derangements caused hepatic injury to promote elevations in circulating FABP1 levels, or whether FABP1 directly worsened LV diastolic function and hemodynamics during exercise. It has been shown that FABP1 is an effective endogenous cytoprotectant, minimizing hepatocyte oxidative damage^27^. The elevation of circulating FABP1 may represent a compensatory mechanism to counteract oxidative stress and inflammation in the liver^21^. Based on these data and ours, we speculate that systemic venous congestion secondary to the elevation in right heart pressures may lead to hepatocyte injury to promote up-regulation of FABP1. Further studies are warranted to determine the mechanisms underlying hepatic injury in HFpEF.

### Diagnostic implications

Diagnosis of HFpEF in people presenting with chronic dyspnea is challenging^6,7,10^. Assessments of clinical characteristics, chest radiography, echocardiography, and blood biomarkers play an important role in the diagnostic evaluation of HFpEF. Natriuretic peptides are the most commonly-used blood-based biomarker to facilitate diagnosis of HFpEF and a recent guideline statement from the ESC has proposed a scoring system based upon echocardiographic markers of diastolic function as well as natriuretic peptides to determine whether HFpEF is present^7^. However, there are well-known limitations of natriuretic peptides, such as an underestimation in obese patients^28–30^. This makes the identification of novel biomarkers that relate to greater elevations of cardiac filling pressures or the presence of HFpEF high priority. It is therefore noteworthy that FABP1 levels were elevated in patients with HFpEF compared to controls, were well correlated with echocardiographic markers of elevated cardiac filling pressures and PA pressures during exercise, and predicted the presence of HFpEF. Although the diagnostic ability of FABP1 was lower than NT-proBNP, FABP1 had an incremental value to identify HFpEF from control subjects over the established H_2_FPEF score. The current data suggest that FABP1 could be a candidate biomarker to help identify HFpEF among patients with chronic dyspnea. Further large-scale studies are required to validate these findings and establish a cut-off for FABP1 levels to allow for incorporation into current diagnostic practice.

### Limitations

This is a single-center study from a tertiary referral center. All participants were referred for exercise stress echocardiography for the evaluation of unexplained exertional dyspnea, introducing selection and referral bias. Although the current study and our previous one both focused on FABP1 levels in HF, the two studies are essentially different in three main perspectives: the aim, study design, and population. The primary aim of the previous study was to determine the prognostic value of FABP1 in HF^23^. In other words, the previous study was a longitudinal outcome study in design. On the other hand, the present study was a cross-sectional study to investigate whether FABP1 levels would correlate with echocardiographic markers of intracardiac pressures during supine bicycle exercise. Regarding the population, the previous study included HF patients regardless of EF and control subjects who were referred to coronary angiography. On the other hand, the present study included HFpEF patients and control subjects who were referred to exercise stress echocardiography for the evaluation of unexplained dyspnea. The pathophysiologic role of FABP1 in HF with reduced EF was beyond our scope. Patients with liver disorders were excluded from the analysis based on liver enzymes. Liver sonographic data were available in six patients, in which two had mild chronic hepatitis and one had mild fatty liver. We cannot exclude the possibility that some patients who did not undergo liver sonography might have had a liver disease that was not evident from liver enzymes^31^. The presence of hepatitis could have biased the results although key results remained similar even after excluding the three patients. The control group was not normal as they were referred for exercise stress echocardiography in the evaluation of exertional dyspnea and had comorbidities such as hypertension and interstitial pneumonia and relatively higher FABP1 levels than healthy controls, which could also bias the results^22^. However, the fact that the control population was more diseased than a truly normal healthy control population only biases our data toward the null. Given the presence of exertional dyspnea and comorbidity burden, control subjects might be considered as pre-HFpEF, and the inclusion of controls might add greater insight into the continuous relationships between the magnitude of FABP1 elevations and cardiac abnormalities across the spectrum from risk to frank HFpEF. We cannot conclude that these observations were specific to HFpEF, or may be observed in other disorders that cause right heart pressures, such as HF with reduced EF or non-Group II pulmonary artery hypertension. Further studies are required to address this question. The small sample size of this study does not allow to simultaneously adjust multivariable factors to analyze the diagnostic power of FABP1 to distinguish HFpEF from controls.

### Conclusions

Serum FABP1 levels are elevated in early HFpEF and the magnitude of elevation is associated with echocardiographic markers of elevated LV filling pressure and PA pressures, systemic congestion, and lower workload. The present study suggests that FABP1 may serve as a potential hepatic biomarker that associates with hemodynamic perturbation, lower cardiac output, and reduced exercise capacity in HFpEF, and that FABP1 may help distinguish HFpEF among subjects with dyspnea. Further studies are required to confirm the current findings.

## Material and methods

### Study population

This was a cross-sectional study that assessed the association between serum FABP1 levels and Doppler echocardiographic hemodynamics at rest and during supine bicycle exercise. We prospectively enrolled consecutive subjects who were referred to our echocardiographic laboratory for exercise stress echocardiography for the evaluation of unexplained exertional dyspnea between November 2019 and June 2020. The study was approved by our Institutional Review Board (Gunma University Hospital, Clinical Research Review Board; IRB2019-047) and was performed in accordance with the Declaration of Helsinki. Written informed consent was obtained by all participants. HFpEF was defined by typical clinical symptoms (exertional dyspnea), normal LV EF (≥ 50%), and objective evidence of elevated left heart filling pressures at rest and/or with exercise: (1) the European Society of Cardiology (ESC) and American Society of Echocardiography/European Association of Cardiovascular Imaging (ASE/EACVI)-recommended echocardiographic diastolic dysfunction; (2) the E/e’ during exercise > 15; or (3) invasively-measured pulmonary capillary wedge pressure [PCWP] at rest > 15 mmHg and/or with supine ergometry exercise ≥ 25mmHg^4,7,10,32^. Subjects who were referred to exercise echocardiography were also screened as a comparator group (non-cardiac dyspnea: controls). Control subjects were required to have (1) exertional dyspnea and (2) no objective evidence of elevated left heart filling pressures at rest and with exercise (criteria above). Subjects with EF < 50%, significant left-sided valvular heart disease (> moderate regurgitation, > mild stenosis), infiltrative, restrictive, or hypertrophic cardiomyopathy, non-Group II pulmonary artery hypertension or exercise-induced pulmonary hypertension without elevation of E/e’ (mPAP with exercise > 30 mmHg with a total pulmonary resistance [i.e., mPAP/CO] of > 3 mmHg min/L)^33,34^, and significant liver disorders (any acute or chronic liver diseases, defined by serum levels of transaminases more than three times the upper limit of normal) were excluded. There was no overlap with our previous study focusing on the prognostic value of FABP1 in HF patients regarding the study subjects^23^. The data underlying this article will be shared on reasonable request to the corresponding author.

### Biomarker measurements

Venous blood samples were obtained just before the assessment of exercise stress echocardiography. Serum FABP1 levels were measured using a commercially available enzyme-linked immunosorbent assay (ELISA) kit (abcam, Cambridge, UK). As specified by the manufacturer, the lower limits of detection of serum FABP1 were 9.4 pg/mL. Serum NT-proBNP levels were also determined using another ELISA kit (abcam, Cambridge, UK). Serum hemoglobin, hepatobiliary enzymes, creatinine, glucose, and lipid profiles were measured by routine automated laboratory procedures.

### Transthoracic echocardiography

Comprehensive resting echocardiography was performed by experienced sonographers using a commercially available ultrasound system (Vivid E95, GE Healthcare, Horten, Norway). LV volumes and EF were determined using apical 4-chamber views^35^. LV systolic function was assessed based on the EF and the systolic mitral annular tissue velocity at the septal annulus (mitral s’). LV diastolic function was assessed using the E, e’, and the E/e’ ratio. Left atrial volume was determined using the biplane method of disks. Stroke volume (SV) was determined from the LV outflow dimension and pulse wave Doppler profile. CO was calculated from the product of heart rate and SV. RV systolic function was assessed using TAPSE and TV s’. RA pressure was estimated from the diameter of the inferior vena cava and its respiratory change. PASP was calculated as 4 × (peak tricuspid regurgitation [TR] velocity)^2^ + estimated RA pressure. The mPAP was calculated as 0.61 × PASP + 2^36^.

Subjects underwent supine cycle ergometry echocardiography, starting at 20 W for five minutes, increasing 20 W increments in three-minute stages to subject-reported exhaustion. Echocardiographic images were obtained at baseline and during all stages of exercise. All Doppler measures represent the mean of ≥ three beats. All studies were interpreted offline and in a blinded fashion by a single investigator (M.O.).

### Calculation of the H_2_FPEF score

The H_2_FPEF score is an evidence-based algorithm that incorporates echocardiographic and clinical variables, which has been shown to provide a valid estimate for HFpEF probability^6^. The H_2_FPEF score is based on four clinical parameters (body mass index [BMI] > 30 kg/m^2^ [2 points], treatment with two or more antihypertensive medicines [1 point], atrial fibrillation [AF, 3 points], and age > 60 years [1 point]) and two echocardiographic variables (E/e’ ratio > 9 [1 point] and PASP > 35 mmHg [1 point]). This results in a categorical H_2_FPEF score ranging from 0 to 9.

### Statistical analysis

Data are reported as mean (SD), median (IQR), or number (%) unless otherwise specified. Between-group differences were compared by unpaired t-test, Wilcoxon rank-sum test, or chi-square test, as appropriate. Pearson’s or Spearman’s correlation coefficients were used to assess relationships between two variables of interest, where non-normally distributed data were log-transformed. Multivariable linear regression analysis was used to test whether differences in FABP1 remain significant after adjusting confounding factors. Receiver operating curves (ROC) were constructed to evaluate the diagnostic ability of FABP1. The incremental diagnostic value of FABP1 to the H_2_FPEF score was evaluated by sequential logistic regression analysis using nested models^37^. The change in overall − 2 log likelihood ratios of the models was used to assess the increment in diagnostic information. All tests were two-sided, with a value of *p* < 0.05 considered significant. All statistical analyses were performed with JMP 14.0.0 (SAS Institute, Cary, NC, USA).
